# A Study on Dust Storm Pollution and Source Identification in Northwestern China

**DOI:** 10.3390/toxics13010033

**Published:** 2025-01-03

**Authors:** Hongfei Meng, Feiteng Wang, Guangzu Bai, Huilin Li

**Affiliations:** 1 Key Laboratory of Cryospheric Science and Frozen Soil Engineering, Northwest Institute of Eco-Environment and Resources, Chinese Academy of Sciences, Lanzhou 730000, China; menghf@llas.ac.cn (H.M.); lihuilin@lzb.ac.cn (H.L.); 2 Key Laboratory of Ecological Safety and Sustainable Development in Arid Lands, Northwest Institute of Eco-Environment and Resources, Chinese Academy of Sciences, Lanzhou 730000, China; baigz@llas.ac.cn; 3 Northwest Institute of Eco-Environment and Resources, Chinese Academy of Sciences, Beijing 100049, China

**Keywords:** Northwestern China arid region, dust storm, aerosol optical depth, transport pathway, dust source region, dust storm impact and mitigation

## Abstract

In April 2023, a major dust storm event in Lanzhou attracted widespread attention. This study provides a comprehensive analysis of the causes, progression, and dust sources of this event using multiple data sources and methods. Backward trajectory analysis using the HYSPLIT model was employed to trace the origins of the dust, while FY-2H satellite data provided high-resolution dust distribution patterns. Additionally, the MAIAC AOD product was used to analyze Aerosol Optical Depth, and concentration-weighted trajectory (CWT) analysis was used to identify key dust source regions. The study found that PM_10_ played a dominant role in the storm, and the AOD values during the storm in Lanzhou were significantly higher than the annual average, highlighting the severe impact on regional air quality. Key meteorological conditions influencing the storm’s occurrence were analyzed, including the formation and eastward movement of a high-potential ridge, convection driven by diurnal temperature variations, and surface temperature increases coupled with decreased relative humidity, which together promoted the generation and development of dust. Backward trajectory and dust distribution analyses revealed that the dust primarily originated from Central Asia, western Mongolia, Xinjiang, and Gansu. From the 19th to the 21st, the dust distribution showed similarities between day and night, with a noticeable increase in dust concentration from night to day due to strong vertical atmospheric mixing. To mitigate the impacts of future dust storms, this study highlights both short-term and long-term strategies, including enhanced monitoring systems, public health advisories, and vegetation restoration in key source regions. Strengthening regional and international cooperation for transboundary dust management is also emphasized as critical for sustainable mitigation efforts. These findings are significant for understanding and predicting the causes, characteristics, and environmental impacts of dust storms in Lanzhou and the Northwestern region.

## 1. Introduction

In recent years, the frequency of extreme climate events has increased due to global climate change and human activities [1]. In Northwestern China, dust storms—a typical form of extreme weather—have become a growing concern, both in terms of frequency and intensity. These events not only degrade the air quality and visibility but also pose significant threats to agriculture, water resources, human health, and infrastructure. In particular, high concentrations of particulate matter (PM_10_ and PM_2.5_) during dust storms can exacerbate respiratory and cardiovascular diseases, posing severe health risks to vulnerable populations [2,3]. The increasing severity of dust storms has raised alarms about their long-term impact on the environment and economy, particularly in arid and semi-arid regions like Lanzhou. Due to its unique geographic and topographic characteristics, Lanzhou has emerged as a key area for dust storm research. The city experienced a major dust storm event in April 2023, characterized by dramatic surges in PM_10_ and PM_2.5_ levels, during which the PM_10_ concentrations surged dramatically from 136 µg/m^3^ on 18 April to 1042 µg/m^3^ on 19 April. This underscores the urgency of conducting research into the mechanisms of dust storms and identifying effective control strategies to mitigate their damage.

The current research on dust storms primarily focuses on various aspects: temporal and spatial characteristics [4,5], responses to meteorological factors [6,7], source identification [8], dust emission and deposition [9,10,11], monitoring and prevention [12,13], and transport processes [14,15]. Among these, climate and weather conditions significantly influence dust storms’ occurrence, development, and transport, with wind speed being a decisive factor [16,17,18,19]. In terms of dust sources and transport paths, Yan [20] used quartz oxygen isotope analysis, combined with MODIS satellite remote sensing and backward trajectory models, to explore the source of a dust event in Xi’an. Guan [21] integrated atmospheric particulate matter with dust events, quantifying the impact of different potential source areas on PM_10_ in the Hexi region. Kandakji [22] combined land use and MODIS remote sensing data to quantify the contribution of anthropogenic and natural sources to dust emissions in the western United States.

Understanding and mitigating the impacts of dust storms begins with identifying their sources and transport pathways. Traditional source apportionment methods, such as chemical mass balance or positive matrix factorization, often rely on detailed chemical composition data, which can be costly and time-consuming to collect [22]. In this study, we apply back-trajectory modeling (using the HYSPLIT model) to trace the transport pathways of dust storms and utilize concentration-weighted trajectory (CWT) methods to identify potential dust source regions. We integrate these approaches with satellite-derived aerosol data (e.g., MAIAC AOD and FY-2H dust monitoring products) to provide a comprehensive overview of dust sources and transport dynamics.

In summary, analyzing the entire dust storm process from multiple perspectives and methods is a key direction for future research [23]. A major gap in the existing research is the integration of diverse data sources, particularly the use of advanced remote sensing products like MCD19A2 for aerosol optical depth (AOD) analysis in urban environments during dust storms. We describe this product in detail in Section 2.2.2. AOD is a key indicator of aerosol concentrations and atmospheric pollution, yet no study has utilized MCD19A2 data to analyze the AOD in Lanzhou during a dust storm. This product, with its low data loss rate and advanced algorithms, offers significant potential for improving AOD inversion results, providing more reliable insights into dust storm dynamics. Beyond understanding the mechanisms of dust storms, future research should also focus on their broader impacts, including health risks and economic losses, as well as effective mitigation strategies. Ultimately, this research will provide valuable scientific evidence to inform policy making and environmental governance, not only in Lanzhou but also in other regions facing similar challenges from dust storm events.

## 2. Materials and Methods

### 2.1. Area Overview

**Figure 1:**
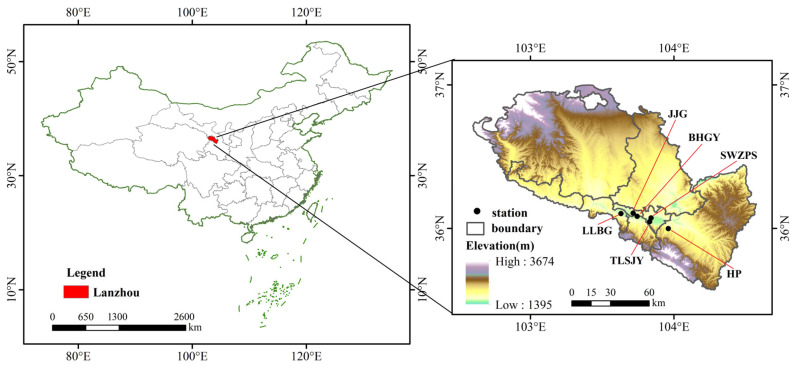
Location of the study area and site distribution map.

Lanzhou, the capital of Gansu Province, lies between 102°36′–104°35′ E and 35°34′–37°00′ N. It is an important industrial base and transportation hub, and a core city on the Silk Road Economic Belt in Northwestern China. The annual precipitation and evaporation are 327.7 mm and 1448 mm, respectively, characterizing a temperate continental climate. Lanzhou is bordered by the Badain Jaran and Tengger deserts to the north, the desert-filled Hexi Corridor to the west, the Qinghai-Tibet Plateau to the southwest, and the Loess Plateau to the east. This makes it a crucial path for dust storms moving eastward in Northwestern China. The city’s unique valley climate (low wind and stable atmospheric layers) also makes it a major deposition area for dust aerosols [24]. This study involves six national monitoring stations from west to east: Lan Lian Bin Guan (LLBG), Jiao Yu Gang (JYG), Bai He Gong Yuan (BHGY), Tie Lu She Ji Yuan (TLSJY), Sheng Wu Zhi Pin Suo (SWZPS), and He Ping (HP) (Figure 1).

### 2.2. Research Data

#### 2.2.1. Station Data

The dust storm lasted from 19 to 21 April 2023. To compare pollutant concentration changes before and after the storm, hourly air pollutant concentration data (PM_2.5_, PM_10_, SO_2_, NO_2_, O_3_, and CO) were collected from six national monitoring stations in Lanzhou from 17 to 23 April. The 17–18 April represented Phase 1, 19–21 April were Phase 2 (the storm phase), and 22–23 April were Phase 3. Daily data were computed from hourly data. The statistical processing and quality control of monitoring data adhered to China’s “Air Quality Monitoring Specifications (Trial)” and “Air Quality Standards (GB 3095-2012)”, ensuring accurate and reliable data.

#### 2.2.2. Aerosol Data

In 2018, NASA (National Aeronautics and Space Administration) introduced the Multi-Angle Implementation of Atmospheric Correction (MAIAC) algorithm for its atmospheric AOD product MCD19A2. Among various AOD products, MAIAC AOD stands out due to its advanced algorithm and having the highest spatial resolution (1 km), which enables aerosol studies at finer scales, such as in urban areas.

Notably, since the release of MAIAC AOD data, numerous scholars have extensively evaluated its applicability. Research has validated not only the accuracy of MAIAC AOD [25,26,27,28] but also its superior performance in arid and semi-arid regions, as well as most urban areas [25,29,30]. These studies consistently demonstrate that MAIAC AOD is reliable and suitable for fine-scale aerosol analysis.

For this study, we utilized the MCD19A2 data version C6 with a wavelength of 550 nm, daily temporal resolution, and a spatial resolution of 1 km × 1 km, covering the period from 17 to 23 April 2023. We processed the data using the Google Earth Engine platform for extraction, cloud removal, projection transformation, and stitching. These processing steps ensured that the dataset met our analysis requirements while maintaining the integrity and quality of the original data.

#### 2.2.3. Meteorological Data

The meteorological data for this study come from two sources. For trajectory calculations, the model requires ARL-format data; hence, the backward trajectory and concentration-weighted trajectory analyses utilized the Global Data Assimilation System (GDAS) meteorological data provided by the National Center for Environmental Prediction (NCEP). These data, accessible at ftp://arlftp.arlhq.noaa.gov/pub/archives/gdas1 (accessed on 7 June 2023), have a spatial resolution of 1° × 1°. Additionally, geopotential height, temperature, relative humidity, wind speed, and wind direction data were sourced from the China Meteorological Data Network (http://data.cma.cn/) (accessed on 5 October 2023) using the Chinese Global Atmospheric Reanalysis (CRA) 40-year product, which has a daily temporal resolution and a horizontal resolution of 34 km.

#### 2.2.4. Dust Data

The Fengyun-2H (FY-2H) satellite was successfully launched on 5 June 2018 and is positioned at 79 degrees eastern longitude. It marked the first time a Chinese geostationary meteorological satellite achieved continuous observation of one-third of the Earth’s surface, ranging from Oceania to Central Africa. The FY-2H satellite provides real-time cloud imagery, clear-sky atmospheric radiation, cloud motion vectors, dust monitoring, and dozens of other remote sensing products. This study utilizes its 9210 dust monitoring product. The product offers hourly resolution and utilizes visible and infrared channel data from the FY-2H satellite. Through algorithmic processing, it provides high-resolution and precise information on dust concentration and distribution.

The FY-2H 9210 dust monitoring product can be accessed online through the National Satellite Meteorological Center (NSMC) of China at https://satellite.nsmc.org.cn/PortalSite/Data/DataView.aspx?currentculture=zh-CN (accessed on 5 October 2023). In this study, we integrated the FY-2H 9210 dust monitoring data with back-trajectory analysis and CWT analysis. This combination allows for a more comprehensive understanding of the origins and transport pathways of the dust storm.

### 2.3. Research Methods

#### 2.3.1. Backward Trajectory Clustering Analysis

This study conducted backward trajectory cluster analysis using the TrajStat software (version 1.5.6) [31], which utilizes the computational module from NOAA’s HYSPLIT model [32]. The HYSPLIT model has been widely applied for identifying sources of pollutants such as dust, due to its ability to simulating atmospheric transport and dispersion processes. In this research, the angle distance algorithm was employed to classify the air flow trajectories reaching Lanzhou, thereby identifying the sources of dust during the storm period. The selection of starting heights and back-trajectory duration were based on previous studies [33,34,35], where the 72 h back-trajectory time frame was chosen because it represents a typical duration for regional dust transport, allowing for coverage of potential long-distance source areas. The starting heights of 500 m, 1000 m, and 1500 m above ground level were selected to encompass the primary layers through which dust and aerosols are transported.

Backward trajectory clustering involves calculating the spatial variance (*SPVAR*) and total spatial variance (*TSV*) for each pair of trajectory combinations to group all air mass trajectories arriving at the target region. The *SPVAR* calculation is shown in Formula (1):

(1) $$\begin{array}{c} SPVAR=\sum_{j=1}^{x}\sum_{i=1}^{t}D_{ij}^{2} \end{array}$$

In this formula, *D_ij_* represents the distance from the *j*-th hour’s position of the *i*-th trajectory to the corresponding point on the average trajectory; *t* is the time index that corresponds to each hourly position along a trajectory. Specifically, for each trajectory, *t* ranges from 1 to the total number of hours in the trajectory. The value of *t* ensures that we are comparing positions at the same relative time points across different trajectories; *x* is the number of trajectories in the cluster; and *TSV* is the sum of *SPVAR* for all clusters.

#### 2.3.2. Concentration-Weighted Trajectory Analysis

Concentration-weighted trajectory (CWT) analysis is a method used for identifying source areas and gridding them [36]. In this study, a grid resolution of 0.1° × 0.1° was selected. Daily concentration data of PM_2.5_ and PM_10_ were used to calculate the weighted values of these particles for each backward trajectory, reflecting the magnitude of PM_2.5_ and PM_10_ along different trajectories. In the CWT analysis, each grid cell contains weighted values for PM_2.5_ and PM_10_, calculated by averaging the PM_2.5_ and PM_10_ values of all trajectories passing through that grid, as determined by Formula (2). When the number of intersections per grid (*n_ij_*) is small, there is significant uncertainty. To minimize this uncertainty, a weighting function is introduced, as outlined in Formula (3).

(2) $$\begin{array}{c} C_{ij}=\frac{\sum_{k=1}^{m}C_{k}T_{ijk}}{\sum_{k=1}^{m}T_{ijk}}\times W\left(n_{ij}\right) \end{array}$$

(3) $$W\left(n_{ij}\right)=\left\{\begin{array}{ll} 1.00 & 80<n_{ij}\\ 0.70 & 20<n_{ij}\leq 80\\ 0.42 & 10<n_{ij}\leq 20\\ 0.05 & n_{ij}\leq 10 \end{array}\right.$$

*C_ij_* represents the weighted concentration in the grid cell at row *i* and column *j*; *C_k_* denotes the pollutant concentration on the *k*-th trajectory; *T_ijk_* indicates the residence time or frequency of the *k*-th trajectory passing through the grid cell at row *i* and column *j*; and m is the total number of trajectories.

#### 2.3.3. GEE

Google Earth Engine (GEE) is a planetary-scale platform for Earth science data and analysis. Traditional remote sensing data processing involves downloading data before preprocessing; however, GEE allows users to develop their own algorithms on the platform, directly utilizing a vast array of datasets including MODIS, Landsat, Sentinel, and others. By leveraging Google’s powerful computing capabilities, GEE significantly reduces the data processing time, lowers usage complexity, and increases efficiency. It has been widely applied in fields such as ecological monitoring, environmental pollution control, and climate change research. In this study, the GEE Code Editor was used to perform data extraction, projection transformation, mosaicking, cropping, and compositing to preprocess the needed data. Subsequently, ArcMap was used for mapping to determine the spatial distribution of AOD.

## 3. Results and Analysis

### 3.1. AOD Spatial Distribution During Severe Pollution in Lanzhou

**Figure 2:**
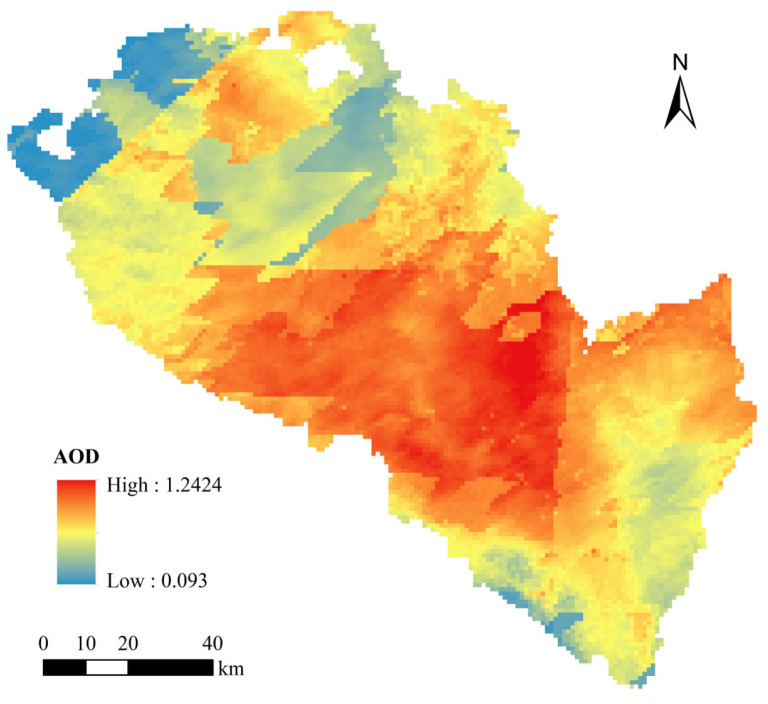
Mean spatial distribution of AOD in Lanzhou from 17 to 23 April 2023.

The spatial distribution of the average AOD in Lanzhou from 17 to 23 April 2023 is depicted in Figure 2. Combining this with the terrain map in Figure 1, it is evident that high AOD values largely coincide with low-elevation areas. This is primarily due to the surrounding mountains that encircle these low-altitude regions. During dust storms, dust aerosols accumulate in these areas and are hindered from dispersing by the mountainous topography, leading to localized airflows and, consequently, higher aerosol optical thickness values. Additionally, these low-elevation areas encompass the main urban districts and surrounding counties of Lanzhou, characterized by high population densities and significant human activities such as traffic, industrial, and agricultural activities. The dense urban development further restricts aerosol dispersion. Studies have shown that the average AOD in Lanzhou from 2011 to 2022 was 0.278 [37]. However, during the study period in 2023, the average AOD increased to 0.772, significantly above the annual average, with the highest recorded value being 1.2424. This highlights the pronounced impact of dust aerosols on optical thickness.

In contrast, the lowest AOD values are observed in less frequented forested mountain regions such as the Qilian Mountains in the northwest of Yongdeng County and Xinglong Mountain in the southeast of Yuzhong County. These high-altitude areas benefit from better aerosol dispersion due to their elevation, which allows pollutants to disperse more freely without being trapped by urban pollution sources. The protective nature of the terrain and vegetation cover minimizes the dust accumulation, resulting in relatively lower AOD values. Moreover, these regions experience minimal human activity, further reducing anthropogenic emissions and maintaining a cleaner air quality compared to urban centers.

### 3.2. Air Quality Changes During a Dust Storm in Lanzhou

The Air Quality Index (AQI) is divided into six levels. By combining data from Table 1 and Table 2, it is evident that during the study period, particularly from 19 to 21 April, the pollution levels were high. On 19 and 20 April, the AQI values reached 500, categorized as Level VI, which corresponds to a “Hazardous” level of health concern.

**Figure 3:**
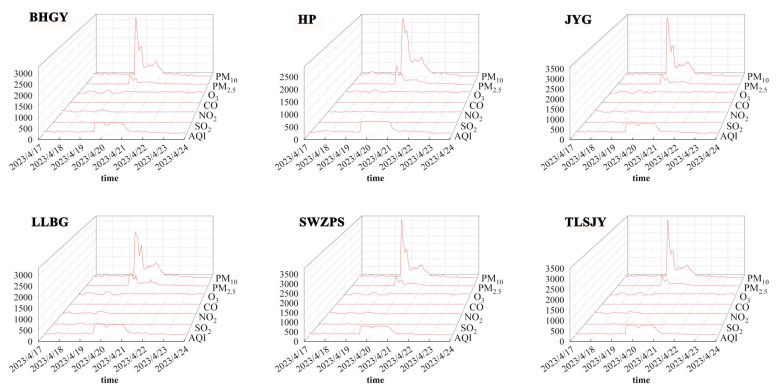
Temporal variation in pollutants (PM_10_ [µg/m^3^], PM_2.5_ [µg/m^3^], CO [mg/m^3^], O_3_ [µg/m^3^], SO_2_ [µg/m^3^], NO_2_ [µg/m^3^]) at six monitoring stations in Lanzhou (LLBG: Lan Lian Bin Guan; JYG: Jiao Yu Gang; BHGY: Bai He Gong Yuan; TLSJY: Tie Lu She Ji Yuan; SWZPS: Sheng Wu Zhi Pin Suo; HP: He Ping) from 17 to 23 April 2023.

Figure 3 displays a time series graph of the AQI and pollutant concentrations for six study sites during the research period. All sites experienced a significant peak in pollution from 19 to 21 April, with notably high concentrations of AQI, PM_10_, and PM_2.5_, indicating that particulate matter was the primary contributor to this severe pollution event. The pollution levels were slightly lower on 21 April and then generally decreased thereafter. The consistent trends in PM_2.5_ and PM_10_ concentrations across all sites indicate the dust storm’s broad and rapid development. Changes in other pollutants such as O_3_, CO, NO_2_, and SO_2_ were not as pronounced as those in particulate matter but varied across different monitoring points.

Table 2 shows the AQI and concentrations of several major pollutants in Lanzhou from 17 to 23 April, as well as the temperature, humidity, and wind speed during the same period. The data are averaged from daily data that were collected at six sites in Lanzhou. The ratios of PM_2.5_ to PM_10_ on 19 and 20 April were 22.84% and 25.54%, respectively, both below 30%, indicating that the dust storm was predominantly driven by PM_10_ [38,39]. From 17 to 20 April, temperatures were above 10 °C, and the relative humidity was below 30%, creating favorable conditions for the generation and spread of the dust storm. Higher wind speeds from 19 to 21 April further contributed to the accumulation of pollutants. Starting on 21 April, changes in meteorological conditions, including a drop in temperature and a significant increase in humidity, facilitated the dilution and dispersion of pollutants, leading to a noticeable decline in the AQI and particulate matter concentrations.

### 3.3. Backward Trajectory of the Dust Storm

**Figure 4:**
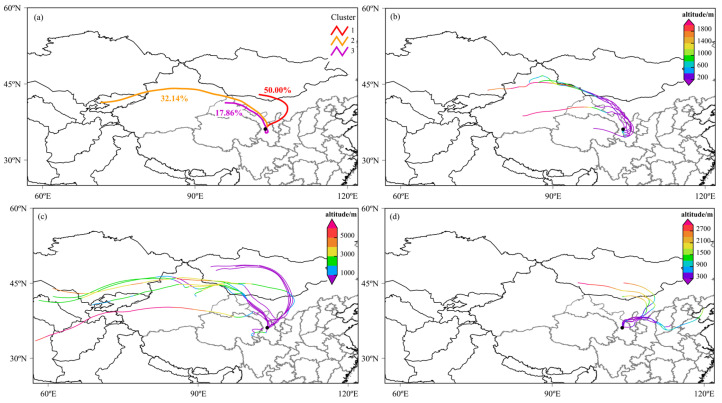
Backward trajectories of atmospheric pollutants from 17 to 23 April 2023: (**a**) overall cluster distribution, (**b**) trajectory directions for 17 to 18 April, (**c**) trajectory directions for 19 to 21 April (dust storm phase), and (**d**) trajectory directions for 22 to 23 April.

Figure 4 illustrates the backward trajectories of atmospheric pollutants from 17 to 23 April. Overall, as shown in Figure 4a and Table 3, the backward trajectory clustering of the atmospheric pollutants during this period can be divided into three groups, with 14, 9, and 5 trajectories in each cluster, respectively. Cluster 1 contains the most trajectories, while Cluster 2 exhibits higher average concentrations of PM_2.5_ and PM_10_. Collectively, Clusters 1 and 2, with their higher trajectory counts and particulate concentrations, represent the primary sources of dust for this storm. The main pathways of these dust particles include central and western Mongolia, northern Ningxia, the five Central Asian countries (Kazakhstan, Kyrgyzstan, Tajikistan, Uzbekistan, and Turkmenistan), the Junggar Basin, and the border regions between Gansu and Inner Mongolia.

Trajectory Directions and Relation to Pollutant Levels: On the 17–18 April (Figure 4b), the air pollutants primarily originated from Xinjiang, central and eastern Qinghai, and the western regions of Inner Mongolia and the Hexi Corridor in Gansu. The west-to-east movement of the trajectories during this period corresponds to the relatively lower pollution levels at monitoring stations, as these source regions contributed less intense dust emissions compared to later periods. This reflects the initial phase of the storm’s development, with limited the transport of high concentrations of particulates. On the 19–21 April (Figure 4c), during the peak pollution phase, the pollutants mainly came from the five Central Asian countries, western Mongolia, and parts of Xinjiang, Gansu, and Inner Mongolia. These trajectories were more dispersed, covering a wider geographic area and indicating diverse and significant sources of dust. This coincides with the observed hazardous PM_10_ concentrations (up to 1042 µg/m^3^, as shown in Table 2) and elevated PM_2.5_ (up to 238 µg/m^3^, as shown in Table 2) levels across the monitoring stations. The diverse sources and strong dust emissions from these regions substantially contributed to the severe air quality degradation observed during this period. On the 22–23 April (Figure 4d), the pollutants primarily originated from southern Mongolia, central Inner Mongolia, Ningxia, northern Shaanxi, and southern Shanxi. This corresponds to a decline in pollutant levels, as shown by the decreasing PM_10_ and PM_2.5_ concentrations that were recorded during this period (Table 2). The reduced dust contributions from these regions and the dissipation of the storm contributed to the observed improvement in air quality.

Trajectory Heights: On 17–18 April, the trajectory heights were relatively low, mostly between 200 and 1400 m, indicating that pollutants were mainly transmitted in lower atmospheric layers. During the dust storm from 19 to 21 April, the height range expanded, with trajectories from 300 m at ground level up to 3000 m, and some even reaching 5000 m. This suggests that pollutants were carried to higher atmospheric layers due to the dust storm’s strong vertical mixing. On 22–23 April, the trajectory heights decreased again, mostly to between 300 and 2700 m, with none exceeding 3000 m, suggesting that the storm had weakened and the pollutant dispersion had decreased. Overall, from 17–18 April to 19–21 April, the average trajectory height increased, showing the storm’s strong vertical mixing, which lifted ground dust to higher atmospheric layers. By 22–23 April, the heights decreased again, indicating that the pollutants had concentrated back in the lower atmosphere.

### 3.4. Potential Pollution Sources During the Dust Storm

**Figure 5:**
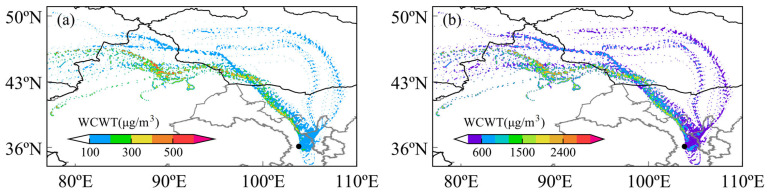
Concentration-weighted trajectory (CWT) analysis of PM_2.5_ and PM_10_ during 19–21 April 2023: (**a**) PM_2.5_ contribution from source regions and (**b**) PM_10_ contribution from source regions. Black dots in the figure represent the location of Lanzhou City.

This study conducted a CWT analysis for PM_2.5_ and PM_10_ during the second phase of the study, as shown in Figure 5. It was found that PM_2.5_ and PM_10_ exhibited similar distribution patterns. High WCWT values were found in southwestern Mongolia, the Junggar Basin in Xinjiang, and central-western Inner Mongolia, identifying these regions as primary dust sources. Both figures collectively demonstrate that during the dust storm, the larger particles (PM_10_) had a broader impact range and higher concentrations. This observation aligns with the characteristics of larger particulate matter spreading with the wind during dust storms.

### 3.5. Meteorological Conditions During the Dust Storm

**Figure 6:**
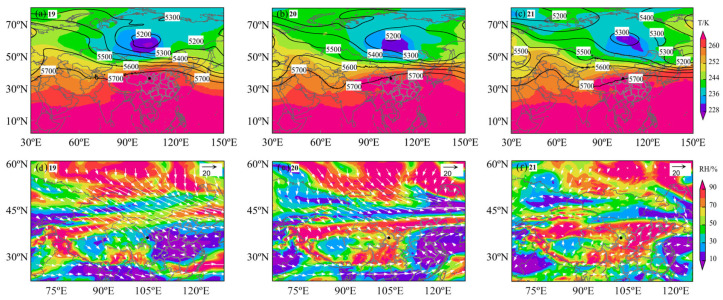
Temperature and 500 hPa geopotential height field (**a**–**c**) and relative humidity and wind field ((**d**–**f**), White arrows indicate wind speed and direction) from the 19th to the 21st. Black dots in the figure represent the location of Lanzhou City.

The distribution of geopotential height and temperature from 19 to 21 April (Figure 6a–c) shows a significant high-pressure ridge (from yellow to red areas) covering the airspace over Northwestern China. This pattern is typically associated with subsiding airflows, which are conducive to the formation of dust storms. Over time, the center of the geopotential height (purple area) moved eastward, suggesting that the eastward movement of the weather system has a direct impact on the occurrence of dust storms. On 19 April, Lanzhou was located near the southwest side of the high-pressure ridge, and by 21 April, it was directly under this ridge. Influenced by the subsiding airflows, this condition led to an increase in local atmospheric pressure, subsequently reducing the humidity and creating dry conditions that favor the formation and suspension of dust. The temperature distribution indicates that the temperatures in Lanzhou were high (red areas), and considering that these measurements were taken at high geopotential heights, the surface temperatures might have been even higher, providing sufficient heat to lift the surface dust into the air. This contributes to the aridity and instability of the ground, which are favorable conditions for the onset of dust storms.

The distribution of the relative humidity and wind speed (Figure 6d–f) shows that during the dust storm, the humidity in Lanzhou was relatively low, with a relative humidity of around 10% on 19 April. Although there was a slight increase in humidity on 20–21 April, Lanzhou remained at the relatively dry center compared to its surrounding areas. This low humidity facilitates the suspension of dust particles.

The wind direction arrows primarily pointed east or southeast, indicating that the Lanzhou area was influenced by westerly or northwesterly winds. Westerlies are a common wind direction in Northwestern China, often intensifying during the spring, and are capable of carrying dust from desert areas eastward or southeastward. The wind speeds on 19 April appeared to be stronger than on 20 and 21 April. This may suggest a direct correlation between the occurrence of dust storms and an increase in wind speed, especially if the wind speed is higher during the initial stages of a dust storm. By analyzing the wind speed and direction data, it is evident that from 19 to 20 April, Lanzhou experienced strong winds from the northwest, which helped lift and transport dust from arid desert regions. On 21 April, the wind direction shifted from northwest to southwest, leading to a gradual decrease in dust from the northwest and the beginning of a reduction in pollution levels.

### 3.6. Dust Conditions During the Dust Storm

**Figure 7:**
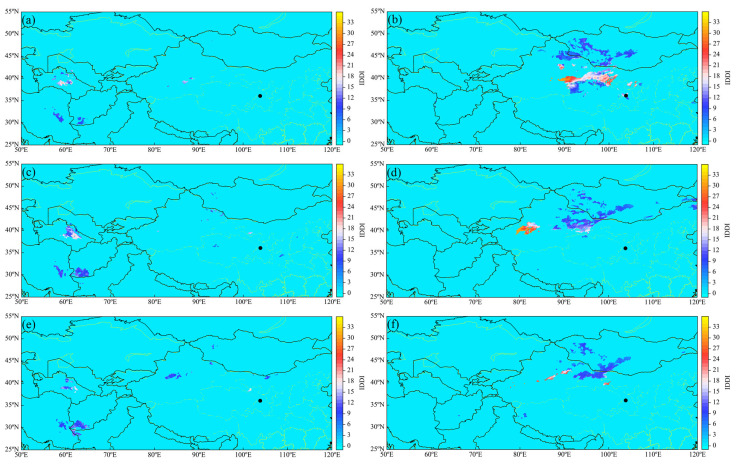
Sand and dust distribution maps from the night of the 19th to the 21st (**a**,**c**,**e**) and during the day (**b**,**d**,**f**). Black dots in the figure represent the location of Lanzhou City.

Figure 7 displays the distribution of dust across Northwestern China during the nights and days of 19–21 April, at midnight (00:00) and noon (12:00). The vertical axis represents the Infrared Difference Dust Index (IDDI), where higher IDDI values indicate greater dust concentrations in the region. The color gradations in the image indicate the density of dust, with warmer colors signifying higher concentrations. The following can be observed: From 19 to 21 April, there was a noticeable increase in the dust concentration from night to day. This phenomenon is primarily due to solar radiation heating the ground during the day, which enhances the temperature difference between the ground and the air, leading to upward air currents. These updrafts can lift dust particles into higher atmospheric layers, increasing the concentration of dust that is suspended in the air. Additionally, the air is typically drier during the day with lower humidity, which facilitates the lifting and spreading of dust.

The trend of dust moving eastward from night to day is apparent, affecting Lanzhou and its surrounding areas. There are two main reasons: First, meteorological maps show a high-pressure ridge above the study area moving eastward, typically associated with larger-scale weather systems like the westerlies. As these systems move eastward, they carry matter along the lower atmospheric layers, including dust particles. Second, during the day, increased surface temperatures cause updrafts and more intense convection, lifting dust into higher atmospheric layers, with stronger westerly winds during the day accelerating the eastward movement of the dust.

Both night and day show a similar distribution pattern, which is related to the persistent weather patterns. During the dust storm, a high-pressure ridge persisted, and the center of the geopotential height remained relatively stable, leading to subsiding airflows that may promote the suspension of dust and maintain a similar distribution pattern both at night and during the day. It can be seen that at night, the dust is mainly distributed in nearby Central Asian countries such as Turkmenistan, Afghanistan, and Iran. During the day, the dust is primarily found in southwestern Mongolia, the Xinjiang and Hexi regions of Gansu, northwestern Qinghai, central and western Inner Mongolia, and northern Ningxia. These regions are located in arid and semi-arid zones, with sparse vegetation cover and loose soil, making it easy for the wind to lift and form dust.

Over the three days, there was a decreasing trend in dust concentration and extent, especially during the daytime. By 21 April, the dust storm was nearing its end, with both the duration and intensity diminishing. Table 1 indicates that the levels of PM_2.5_ and PM_10_ in Lanzhou were gradually decreasing. As shown in Figure 5, the wind strength on 21 April was lower than on the previous two days, and the wind direction shifted from northwest to southwest. With the reduction in dust concentration at the source and the weakening and change in wind direction, the concentration of dust further decreased.

The dust distribution observed in Figure 7 complements the backward trajectory analysis by showing significant dust concentrations along the transport pathways that were identified by HYSPLIT. Moreover, the high-value areas of dust distribution closely match the WCWT results, providing mutual validation of the dust storm’s source regions and pathways.

## 4. Conclusions

Through a comprehensive analysis of the major dust storm event in Northwestern China in April 2023, the following conclusions have been drawn:

The Significance of AOD and Dominance of PM_10_: During the dust storm, the AOD values in Lanzhou were significantly above the average, underscoring the substantial impact of the increased dust aerosol concentration on atmospheric transparency. Furthermore, the marked increase in particulate matter concentrations confirmed their role as the primary pollutants, with the ratio of PM_10_ to PM_2.5_ further emphasizing the dominant role of PM_10_ in such events.

Combined Impact of Adverse Meteorological Conditions: By comparing meteorological parameters such as the geopotential height, temperature, and humidity, this study has revealed the unfavorable meteorological backdrop for the formation of dust storms. The formation and subsequent eastward movement of a high-pressure ridge facilitated the ascent and suspension of dust particles. In Lanzhou, rising temperatures and decreasing relative humidity further promoted the formation and accumulation of dust, intensifying the strength and extent of the dust storm.

Identification of Dust Sources: Combining backward trajectory analysis and concentration-weighted trajectory analysis, the main sources of dust were identified. The pollutants in this dust storm were transported to Lanzhou by northwesterly winds from Central Asia, central and western Mongolia, and regions within China such as Xinjiang and Gansu. The arid environments and geographical locations of these areas are the primary sources of dust particles.

Differences in Day and Night Dust Distribution: From 19 to 21 April, there was a noticeable increase in the dust concentration from night to day. This increase was primarily due to solar radiation heating the ground during the day, which enhanced the temperature difference between the ground and the air, leading to the formation of updrafts. These updrafts carried dust particles into higher atmospheric layers, increasing the concentration of dust in the air. The dry air conditions during the day also facilitated the lifting and dispersion of dust. The dust distribution showed some similarities between day and night. At night, the dust was primarily concentrated in nearby Central Asian countries such as Turkmenistan, Afghanistan, and Iran. During the day, it was mainly distributed in southwestern Mongolia, Xinjiang, the Hexi region of Gansu, northwestern Qinghai, central and western Inner Mongolia, and northern Ningxia, which are arid and semi-arid regions.

Short-Term and Long-Term Strategies for Dust Storm Mitigation: To address the impacts of future dust storms, both short-term and long-term mitigation strategies should be considered. Short-term measures include enhancing real-time monitoring and early warning systems by utilizing advanced satellite data, such as that from Fengyun-2H, along with ground-based sensors to provide timely alerts to the public. Public health advisories should be implemented during dust storm events, recommending reduced outdoor activities and the use of protective measures like N95 masks. For long-term measures, it is important to promote vegetation restoration and afforestation in major dust source regions, such as Central Asia, western Mongolia, and the Hexi Corridor, to stabilize the soil and reduce dust emissions. Additionally, strengthening regional and international cooperation for dust source management and mitigation is crucial, especially with countries in Central Asia that significantly contribute to transboundary dust transport.

## Figures and Tables

**Table 1:** AQI categories corresponding to different levels of health concern.

AQI Values	Air Quality Level	Levels of Health Concern	Colors
0–50	I	Good	Green
51–100	II	Moderate	Yellow
101–150	III	Unhealthy for Sensitive Groups	Orange
151–200	IV	Unhealthy	Red
201–300	V	Very Unhealthy	Purple
301–500	VI	Hazardous	Maroon

**Table 2:** Concentration of relevant pollutants (CO unit in mg/m^3^, others in µg/m^3^) and meteorological data (temperature/°C, humidity/%, wind Speed/m/s) from 17 to 23 April 2023.

Date	AQI	PM_2.5_	PM_10_	CO	NO_2_	SO_2_	O_3_	Temperature	Humidity	Wind Speed
04.17	97	44	144	0.6	40	10	114	18.22	21.47	1.88
04.18	93	46	136	0.6	53	16	140	18.68	24.91	1.76
04.19	500	238	1042	0.5	38	10	89	16.01	27.93	3.84
04.20	500	167	654	0.3	12	6	80	10.10	24.42	2.88
04.21	122	68	193	0.4	12	5	85	7.54	53.25	2.58
04.22	69	46	87	0.4	14	6	86	6.93	59.38	2.02
04.23	42	29	32	0.6	18	6	76	6.31	70.95	1.60

**Table 3:** Pollutant information for the three backward trajectory clusters and the regions that they pass through.

Cluster	Number	PM_2.5_		PM_10_		Transit Area
		Mean	Sd	Mean	Sd	
1	14	62.9	49.65	179.63	219.81	Mongolia, the central and western regions of Inner Mongolia, and northern Ningxia
2	9	152.48	125.09	681.3	686.46	Gansu and Inner Mongolia border area and north-central Xinjiang
3	5	44.11	5.74	129.03	18.25	Border regions between Gansu and Inner Mongolia

Sd: standard deviation.

## Data Availability

The data presented in this work are available on request from the first author. Requests for data should be addressed to Hongfei Meng (menghf@llas.ac.cn).
